# Supplementary material for: Methane production by Methanothrix thermoacetophila via direct interspecies electron transfer with Geobacter metallireducens
Source: mBio. 2023 Jun 12;14(4):e00360-23. doi: 10.1128/mbio.00360-23 (PMC10470525; doi:10.1128/mbio.00360-23)
Supplement: Supplemental figures — Figures S1 to S3. [file mbio.00360-23-s0001.pdf]

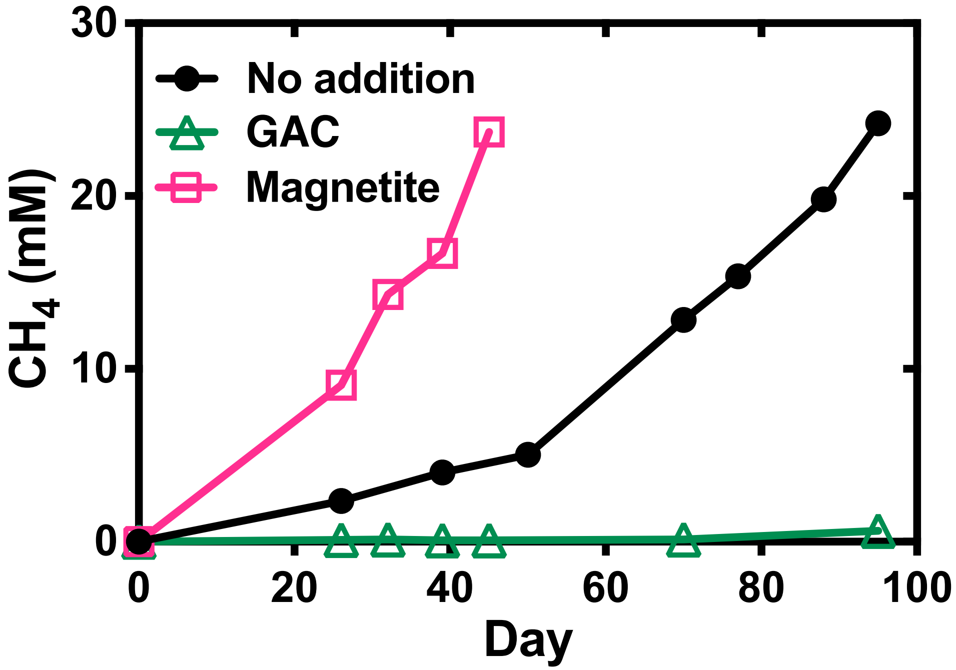

Supplementary Figure S1. Methane productions by *Mx. thermoacetophila* during the first transfer when co-cultures of *G. metallireducens* and *Mx. thermoacetophila* were becoming established with ethanol (20 mM) as the electron donor.

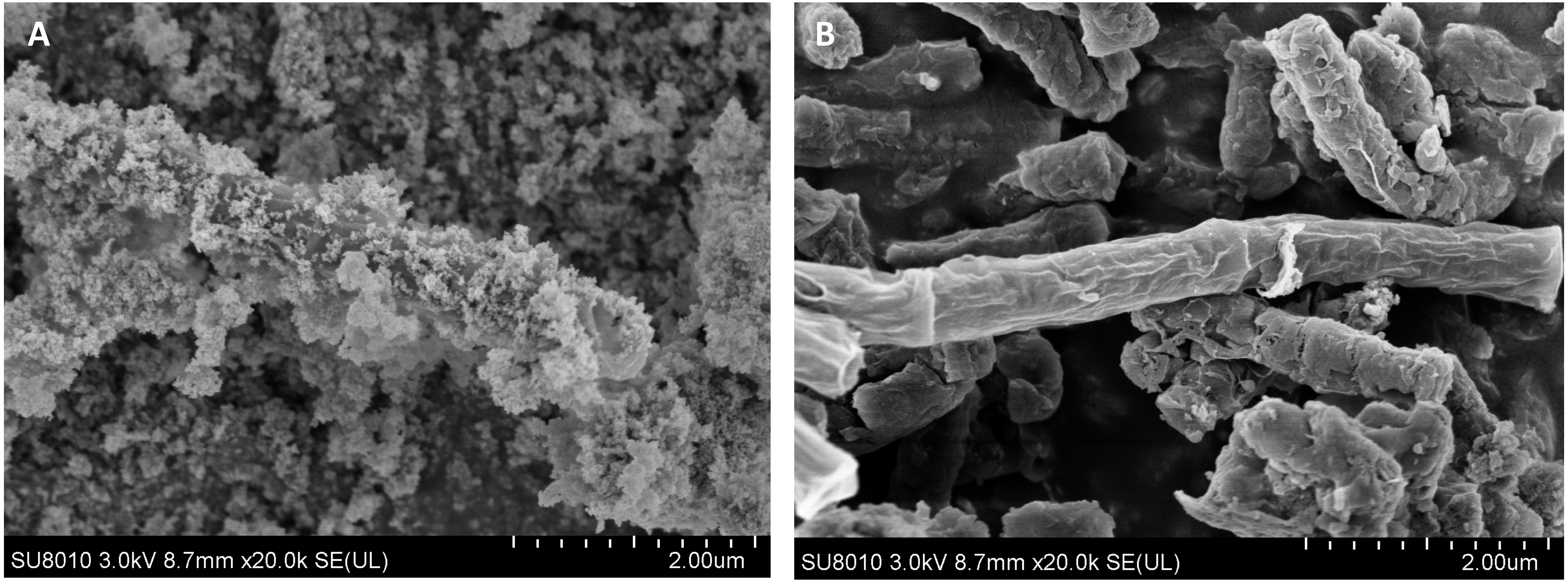

Supplementary Figure S2. Scanning electron microscopy (SEM) images of *Mx. thermoacetophila* in the presence (A) or absence (B) of 10 mM magnetite. Scale bars: 2 µm

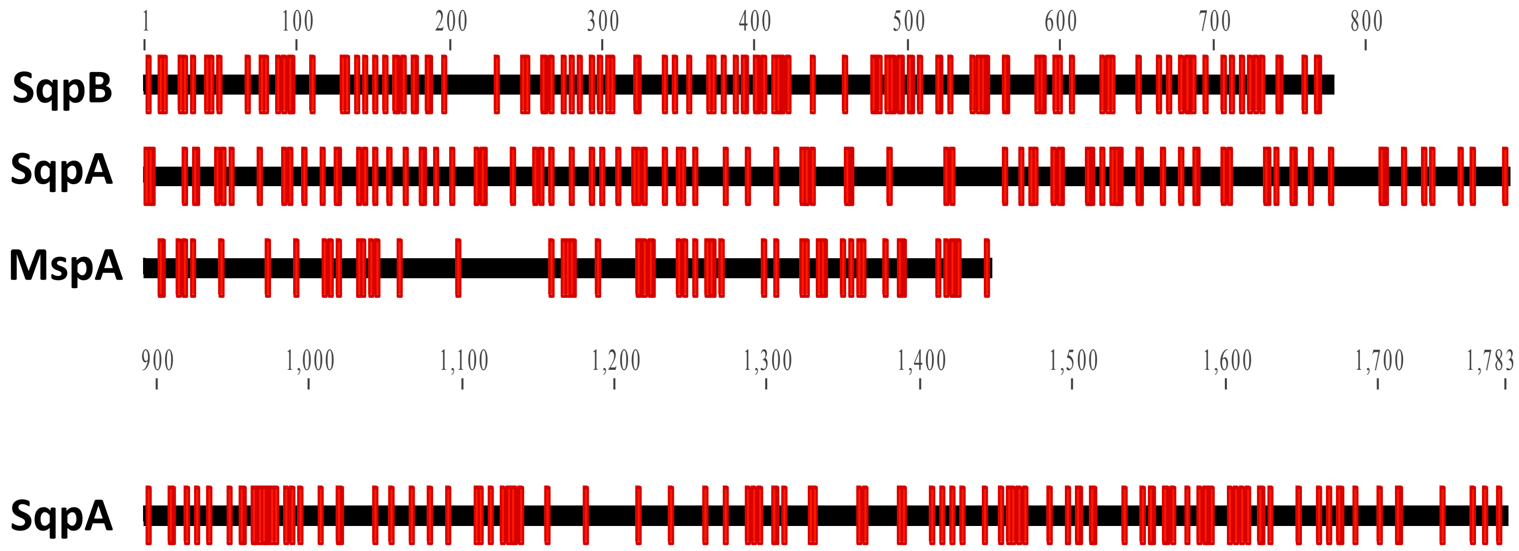

	% aromatic residues	Size of largest gap between aromatic residues
**SqpB**	14.80	33
**SqpA**	11.33	36
**MspA**	10.67	59

Supplementary Figure S3. Arrangement of aromatic amino acid (tryptophan, phenylalanine, tyrosine, and histidine) residues in mature SqpB (Mthe_0877), SqpA (Mthe_0878), and MspA (Mthe_1069) proteins from *Mx. thermoacetophila*. Red rectangles represent aromatic amino acid residues.
