# Supplementary material for: Methane production by Methanothrix thermoacetophila via direct interspecies electron transfer with Geobacter metallireducens
Source: mBio. 2023 Jun 12;14(4):e00360-23. doi: 10.1128/mbio.00360-23 (PMC10470525; doi:10.1128/mbio.00360-23)
Supplement: Supplemental Text — Carbon metabolism of Mx. thermoacetophila grown under various conditions. [file mbio.00360-23-s0006.pdf]

**Supplementary Text**

## Carbon metabolism of *Mx. thermoacetophila* grown under various conditions

Many carbon metabolism genes were highly expressed by *Mx. thermoacetophila* cells grown under both DIET and acetate conditions. In all of the conditions, most genes from pathways for methanogenesis from acetate and $CO_2$, carbon fixation, the reductive citric acid cycle, and carbon monoxide and formate metabolism had RPKM values that were more than 2-fold above the median RPKM values (Figure 6 and Table S3). Addition of magnetite also significantly increased the expression of almost all of these genes (Figure 6 and Table S3).

**Acetoclastic pathway**

Genes coding for proteins from the acetoclastic pathway, acetyl-CoA synthetase (*acs*) and CO dehydrogenase/acetyl-CoA synthase (*cdh*), were highly expressed in all 4 conditions (Figure 6 and Table S3). There are 4 putative acetyl-CoA synthetase (*acsA*) genes in the *Mx. thermoacetophila* genome. Similar to previous reports (1), *acsA-1* (Mthe_1194) was the most highly expressed and had RPKM values that were 105 to 255 ($p$-values < $9.0\times10^{-6}$) times higher than the median. Genes coding for the delta and gamma subunits from the acetyl-CoA decarbonylase/synthase complex (*cdhDE*; Mthe_0287-0288) were also 39 to 133 ($p$-values < 0.002) times more highly expressed than the median RPKM values. Addition of magnetite significantly increased expression of these genes (Figure 6 and Table S3).

**Carbon fixation**

*Methanothrix* species have a RuBisCO-mediated carbon fixation pathway (reductive

hexulose phosphate (RHP) pathway) that forms formaldehyde as an intermediate (2). The *Mx. thermoacetophila* genome also has two genes (Mthe_0988 and Mthe_1603) that code for formaldehyde-activating enzyme (Fae), an enzyme that catalyzes the conversion of formaldehyde into 5,10-methylenetetrahydromethanopterin, an intermediate in the methanogenic $CO_2$ reduction pathway. In addition to duplicate copies of *fae*, the *Mx. thermoacetophila* genome has multiple copies of many of the genes from the RHP pathway, several of which are fusion proteins. One of the *fae* genes is fused to another gene coding for 3-hexulose-6-phosphate synthase (*hps*), which is the enzyme in the RHP pathway that catalyzes formation of formaldehyde and D-ribulose-5-phosphate from D-arabino-3-hexulose-6-phosphate. Other *Methanothrix* species also have *fae/hps* fusion genes, and it has been proposed that this fusion minimizes carbon loss because formaldehyde released from the RHP pathway can be directly fed into the methanogenesis pathway (2).

Similar to studies of *Methanothrix* species participating in DIET in GAC-amended reactors (3), *Mx. thermoacetophila* was highly expressing most of the RHP pathway genes in all conditions (Figure 6 and Table S3). Addition of magnetite significantly enhanced expression of many of these genes. In particular, the gene coding for ribulose-bisphosphate carboxylase (RuBisCO, Mthe_1616), which is the enzyme responsible for the first step in the carbon fixation pathway was 2.7 ($p$-value = $1.04\times10^{-6}$) and 5.6 ($p$-value = $5.38\times10^{-13}$) times more highly expressed in the presence of magnetite when cells were grown by DIET or acetoclastic methanogenesis, respectively (Supplementary Table S4).

Several of the fused genes from the RHP pathway were more highly expressed than the non-fused genes. For example, the *fae/hps* fusion had higher expression levels than either singular gene: *fae/hps* (Mthe_0988) had 2.2 to 5.1 ($p$-values < $2.1\times10^{-5}$) times higher

expression than *fae* (Mthe_1603) and 2.7 to 4.7 higher expression than *hps* (Mthe_0455) (*p*-values < $1.7\times10^{-4}$). Expression of a gene coding for another fusion protein composed of fructose-bisphosphate aldolase and D-fructose 1,6-bisphosphatase (*fbp/aldo*; Mthe_1333) was 6.3 to 9.6 times higher than fructose-bisphosphate aldolase (Mthe_0380) (*p*-values < $1.3\times10^{-5}$).

In addition to the RHP pathway, the genome of *Mx. thermoacetophila* has a gene coding for ATP-citrate lyase (*acly*; Mthe_1476), which can form acetyl-CoA and oxaloacetate from citrate and plays a major role in the reductive TCA cycle (4-6). This gene was 4.6 to 8.1 times higher (*p*-value < 0.003) than the median RPKM values in all of the conditions (Figure 6 and Table S3). High expression of *acly* suggests that acetyl-CoA was present in cells even during growth by DIET, which helps to explain why acetoclastic genes were being expressed at high levels by DIET-grown cells.

**Carbon dioxide reduction pathway**

*Mx. thermoacetophila* is not capable of hydrogenotrophic growth (7) as it does not have any hydrogenase proteins (8) and hydrogenase activity has not been detected in *Mx. thermoacetophila* membranes (9). However, it does have genes that code for proteins from the $CO_2$ reduction pathway, which is the pathway used by hydrogenotrophic methanogens for methane production (8). Previous studies have shown that these genes are highly expressed by DIET-grown *Methanothrix* cells (10, 11). However, transcriptomic comparisons of $CO_2$ reduction genes between DIET-grown and acetoclastic cells were not done in these previous experiments.

The elevated expression of $CO_2$ reduction pathway genes may be explained by the finding that genes involved in the formation of formate and carbon monoxide were also

highly expressed and both of these intermediates feed into the $CO_2$ reduction pathway (Figure 6). *Mx. thermoacetophila* cannot utilize CO or formate as substrates for methanogenesis (7, 12), however, genes coding for formate dehydrogenase and carbon monoxide dehydrogenase are found in the genome. Genes from an operon with two genes coding for the alpha subunit of formate dehydrogenase (*fdhA*; Mthe_0915, Mthe_0913) and an FdhB-like ferredoxin reductase protein (Mthe_0914) with an FAD-binding site that could bind coenzyme $F_{420}$ were highly expressed in all of the conditions; 4 to 7 times higher than median RPKM values ($p$-values < 0.007). The enzyme complex encoded by this gene cluster could potentially oxidize formate to $CO_2$ and transfer electrons to $F_{420}$ to provide a source of reduced $F_{420}$ for methanogenesis.

The genome also has two gene clusters that code for anaerobic carbon-monoxide dehydrogenase complexes (*cdhAB*; Mthe_0291-0292 and *cooSF*; Mthe_1340-1341) that catalyze $CO_2$ formation from carbon monoxide. RPKM values for genes from the CdhAB and CooSF complexes were 52 to 202 ($p$-values < $9.5\times10^{-6}$) and 4 to 14 ($p$-values < 0.002) times higher than median RPKM values (Figure 6 and Table S3).

**Reference**


1. Berger S, Welte C, Deppenmeier U. 2012. Acetate activation in *Methanosaeta thermophila*: characterization of the key enzymes pyrophosphatase and acetyl-CoA synthetase. Archaea 2012:315153.
2. Kono T, Mehrotra S, Endo C, Kizu N, Matusda M, Kimura H, Mizohata E, Inoue T, Hasunuma T, Yokota A, Matsumura H, Ashida H. 2017. A RuBisCO-mediated carbon metabolic pathway in methanogenic archaea. Nat Commun 8:14007.
3. Yang P, Tan GA, Aslam M, Kim J, Lee PH. 2019. Metatranscriptomic evidence for classical and RuBisCO-mediated $CO_2$ reduction to methane facilitated by direct interspecies electron transfer in a methanogenic system. Sci Rep 9:4116.
4. Hugler M, Sievert SM. 2011. Beyond the Calvin cycle: autotrophic carbon fixation in the ocean. Ann Rev Mar Sci 3:261-89.
5. Verschueren KHG, Blanchet C, Felix J, Dansercoer A, De Vos D, Bloch Y, Van Beeumen J, Svergun D, Gutsche I, Savvides SN, Verstraete K. 2019. Structure of

ATP citrate lyase and the origin of citrate synthase in the Krebs cycle. Nature 568:571-5.

6. Hu Y, Holden JF. 2006. Citric acid cycle in the hyperthermophilic archaeon *Pyrobaculum islandicum* grown autotrophically, heterotrophically, and mixotrophically with acetate. J Bacteriol 188:4350-5.
7. Kamagata Y, Mikami E. 1991. Isolation and characterization of a novel thermophilic *Methanosaeta* strain. Int J Syst Evol Microbiol 41:191-6.
8. Smith KS, Ingram-Smith C. 2007. *Methanosaeta*, the forgotten methanogen? Trends Microbiol 15:150-5.
9. Welte C, Deppenmeier U. 2011. Membrane-bound electron transport in *Methanosaeta thermophila*. J Bacteriol 193:2868-70.
10. Rotaru A-E, Shrestha PM, Liu F, Shrestha M, Shrestha D, Embree M, Zengler K, Wardman C, Nevin KP, Lovley DR. 2014. A new model for electron flow during anaerobic digestion: direct interspecies electron transfer to *Methanosaeta* for the reduction of carbon dioxide to methane. Energy Environ Sci 7:408-15.
11. Liu C, Sun D, Zhao Z, Dang Y, Holmes DE. 2019. *Methanothrix* enhances biogas upgrading in microbial electrolysis cell via direct electron transfer. Bioresour Technol 291:121877.
12. Zinder SH, Anguish T. 1992. Carbon monoxide, hydrogen, and formate metabolism during methanogenesis from acetate by thermophilic cultures of *Methanosarcina* and *Methanothrix* strains. Appl Environ Microbiol 58:3323-9.
